# Notch Signaling Is Associated with Pulmonary Fibrosis in Patients with Pigeon Breeder's Lung by Regulating Oxidative Stress

**DOI:** 10.1155/2024/7610032

**Published:** 2024-08-06

**Authors:** Zhichuang Lian, Remila Kuerban, Zongxin Niu, Paruzha Aisaiti, Chao Wu, Xiaohong Yang

**Affiliations:** ^1^ Graduate School Xinjiang Medical University, Urumqi 830001, China; ^2^ Department of Respiratory and Critical Care Medicine People's Hospital of Xinjiang Uygur Autonomous Region, Urumqi 830001, China

## Abstract

This study explored the molecular mechanism underlying the association of Notch signaling and oxidative stress with the occurrence of pulmonary fibrosis in patients with pigeon breeder's lung (PBL). Rat models of fibrotic PBL were constructed with freeze-dried protein powder, and the animals were divided into the control (intratracheal instillation of normal saline; *n* = 9), M (PBL model; intratracheal instillation of freeze-dried protein powder; *n* = 9), and M + D (PBL+ the Notch inhibitor DAPT; *n* = 9) groups. Immunohistochemistry was employed to observe the protein levels of pathway factors and *α*-SMA, and the levels of ROS, GSH-PX, SOD, and MDA were observed using ELISA. To verify the results of the animal experiment, cytological models were constructed. The M group and the M + D group had significantly increased *α*-SMA levels (*P* < 0.05). Although both groups had significantly higher key protein levels in the Notch channel, the M + D group had significantly lower levels relative to the M group (*P* < 0.05). Oxidative stress products were examined, and the levels of MDA and ROS were significantly increased, while those of GSH-PX and SOD were significantly decreased in the M and M + D groups as compared to the control, but the M group and the M + D group significantly differed (*P* <  0.05). These findings were further validated by the cytological experiment. Notch signaling is associated with pulmonary fibrosis in PBL by regulating cellular oxidative stress, and inhibiting this pathway can slow down pulmonary fibrosis progression.

## 1. Introduction

Pigeon breeder's lung (PBL) is a type of hypersensitivity pneumonitis (HP) that is a diffuse interstitial granulomatous pulmonary disease [1]; this condition is characterized by inflammatory changes in the lungs after sensitive individuals repeatedly breathe in pigeon dander and other shed materials during long-term pigeon breeding [2]. PBL accounts for approximately 60% of HP [3].

Pulmonary fibrosis develops with a complex procedure, whereby a variety of cellular signaling pathways and factors participate [4]. The Notch signaling pathway is an important intercellular communication mechanism, which plays a pivotal role in regulating tissue development and determining cell fate [5]. In patients with acute PBL, the secretion of the receptor of the Notch signaling pathway Notch4 increases, and this increase is correlated with the development of pulmonary fibrosis in these patients; this abnormal activation of the Notch pathway might be closely associated with elevated production of oxidative stress products and the formation of fibroblasts [6]. A study on renal fibrosis has shown that activating this pathway can exacerbate oxidative stress and exacerbate renal fibrosis [7]. Compared to other organs, lung tissue is exposed to higher concentrations of oxygen and is therefore more susceptible to oxidative stress damage. When there is excessive production of reactive oxygen species (ROS) or in another way, say, the in vivo counter-ROS capacity is insufficient, ROS will be excessively present; this condition will induce the occurrence of oxidative stress and then result in damage to lung tissue, which is followed by tissue remodeling [8]. Studies have shown that abnormalities in Notch signaling and the presence of oxidative stress are closely associated with the occurrence of pulmonary fibrosis [9–11], but their interaction and mechanism have not yet been completely elucidated.

Type I alveolar epithelial cells (AECIs) are the main component of the alveolar wall, which are mainly responsible for gas exchange [12] and thus play a crucial role in the development of pulmonary fibrosis. AECI injury-induced apoptosis and oxidative stress are important factors in the occurrence of pulmonary fibrosis. Type II alveolar epithelial cells (AECIIs) are another type of alveolar cell that are responsible for gas exchange and the synthesis of pulmonary surfactants. AECII damage can reduce alveolar surfactants, leading to alveolar instability and intensifying inflammatory reactions, thereby inducing pulmonary fibrosis [13, 14]. When lung tissue is damaged, fibroblasts are activated and secrete a large amount of extracellular matrix, resulting in the reconstruction and fibrosis of the alveolar wall. In addition, the formation of fibroblasts and anomalous activation of the Notch channel and oxidative/antioxidant imbalance are important pathways for the occurrence of pulmonary fibrosis [15]. AECIs, AECIIs, and fibroblasts are the main cells in lung tissue, and they play important roles in pulmonary fibrosis. However, the relationship of Notch signaling with oxidative stress in these three target cells, as well as the process through which Notch signals and oxidative stress lead to pulmonary fibrosis via these targets, has not been clarified. A deep understanding of the interaction between cell functions and pathways is the key to further studies on the issues of how pulmonary fibrosis develops and what treatment plans can be effective for this condition.

Based on the aforementioned, although the role of Notch signals and oxidative stress in various diseases has attracted much attention from researchers in recent years [16], the roles of the Notch channel and oxidative stress during PBL development into pulmonary fibrosis have not been clarified. In addition, the interaction between the Notch pathway and oxidative stress in PBL has yet to be determined.

In our previous study, we enrolled 20 fibrotic PBL patients and 20 healthy controls, and the results showed that both the Notch signaling pathway and the fibrosis marker *α*-SMA were highly expressed; the correlation analysis showed a significant positive correlation between them (*r* = 0.898, *P* < 0.05) [17], which indicated that the Notch pathway was closely associated with the development of pulmonary fibrosis. In this study, we moved further to preliminarily explore the mechanism underlying the participation of oxidative stress and Notch signaling in PBL pulmonary fibrosis. To achieve this goal, we first established a rat model from PBL progression to fibrosis-type PBL with the freeze-dried allergen powder made from pigeon shedding [17]. By intervening in the Notch signaling pathway, we observed how rat lung tissue pathologically changed and how the markers of the Notch signaling pathway and oxidative stress were expressed. Then, we established an in vitro cytological model of PBL. After intervening in the Notch pathway in cells, we observed oxidative stress in the model to verify the mechanism whereby the Notch pathway participated in pulmonary fibrosis development by regulating oxidative stress. The findings of this study might offer a new theoretical reference to the prevention and treatment of fibrotic PBL.

## 2. Materials and Methods

### 2.1. Animals

#### 2.1.1. Animals

The healthy clean-grade Sprague Dawley (SD) rats involved in this study were of either sex (Xipur-Bikai, Shanghai, China; license no., SCXK (Hu) 2018-0006). The rats weighed 180 g to 200 g and were aged 6 w to 8 w. T.

All experiments were performed in rigorous accordance with relevant regulations on experimental animals, and this study gained ethical approval from People's Hospital of Xinjiang Uygur Autonomous Region (KY201803715).

#### 2.1.2. Antigen Preparation

The preparation and purification of antigens were first proposed in 1969 and have undergone a number of modifications to date. The method for allergen preparation was as follows: Pigeon droppings were collected and then dissolved in PBS according to a ratio of 1 : 20. After filtration and dewatering for 24 h, the freeze-dried powder was prepared. The active components of pigeon droppings can be preserved to the maximal extent as a freeze-dried powder, which can be preserved at 1–4°C for later use. The current research group successfully produced freeze-dried allergen powder with pigeon droppings that were previously provided by the cooperating unit of Xinjiang Xintou Pigeon Industry Co., Ltd.

#### 2.1.3. Animal Groupings and Treatment

In our previous study, we established rat models of PBL in different pathological stages, which showed that intratracheal instillation of a freeze-dried allergen powder suspension at a dose of 300 *μ*l/20 *μ*g once per week could establish pulmonary fibrosis in rats [17]. In this study, we followed this method. The models in this study were established according to the following grouping method:

1. The control group (*n* = 9): the animals were instilled intratracheally with physiological saline.
2. The M group (*n* = 9): the animals were instilled intratracheally with freeze-dried allergen powder suspension at a one-off dose of 300 *μ*l/20 *μ*g per week for 20 weeks.
3. The M + D group (*n* = 9): the animals were treated in the same way as was done for the M group plus a DAPT (a Notch inhibitor) injection via the tail vein at a dose of 0.05 mg/kg (twice per week). The modeling lasted for 20 weeks.

At 20 w, hematoxylin-eosin (HE) and Masson staining were carried out to observe pulmonary fibrosis. After successful modeling was confirmed, other observations and analyses were performed.

#### 2.1.4. Observational Indices

1. General condition. The general conditions of the rats were observed.
2. HE and Masson staining to determine pathological changes in pulmonary tissue. Left pulmonary tissue was subjected to fixation, dehydration, paraffin embedding, sectioning, dewaxing, HE staining, and Masson staining. Optical microscopy (×200) was utilized to observe the pathological changes in the sections, and the severity was also assessed.
3. Immunohistochemical analysis of the levels of *α*-SMA (pulmonary fibrosis marker), Notch1, Notch2, Jag1, Jag2, DLL1, and DLL4. The paraffin sections were heated at 65°C for 1 h and then washed thrice with PBS. The sections were repaired in EDTA antigen repair solution for 10 min. Endogenous peroxidase was blocked by a 10 min incubation with hydrogen peroxide (3%) at room temperature. After three washes with BPS, the sections underwent BSA (5%) blockage for 20 min. Primary antibodies (100 *μ*L), including anti-Notch1 (Thermo Fisher; dilution, 1 : 100), anti-Notch2 (Thermo Fisher; dilution, 1 : 200), anti-Jag1 (Biorbyt; dilution, 1 : 200), anti-Jag2 (Affinity; dilution, 1 : 100), anti-DLL1 (Affinity; dilution, 1 : 200), anti-DLL4 (Affinity; dilution, 1 : 100), and anti-*α*-SMA (Abcam; dilution, 1 : 200), were applied for incubation at 4°C overnight. After PBS solution removal, the corresponding secondary antibodies (100 *μ*L), including rabbit secondary antibody (Dako Denmark; dilution, 1 : 200) and Rat secondary antibody (Dako Denmark; dilution, 1 : 200), were applied to each section for 30 min incubation at 37°C. After thrice PBS washing, DAB solution (100 *μ*L) was applied to each section, and coloration was microscopically controlled. After complete color development, the specimen was rinsed with distilled water, restained with hematoxylin, and then differentiated with 1% hydrochloric alcohol (1 s). Ammonia was applied to the section to return the color to blue. Afterward, the sections were subjected to dehydration with graded ethanol (70%–100%; 10 min for each gradient), xylene transparency, and neutral gum sealing.
4. Biochemical analysis of the SOD, MDA, and GSH-PX levels in serum and lung tissue and ROS levels in tissue. The kits included an active oxygen kit (S0033; Beyotime, Shanghai, China), SOD kit (S0101; Beyotime), GSH-PX detection kit (A005; Jiancheng, Nanjing), and malondialdehyde testing kit (A003-1; Nanjing Jiancheng). Measurements were carried out according to the methods described in the kits.

### 2.2. Cytology Experiments

#### 2.2.1. Cells

Pulmonary fibroblasts, AECI, and AECII were isolated from healthy adult SD rats.

#### 2.2.2. Cell Groupings and Treatment

Due to the difficulty-generating AECIIs, we purified the three types of cells for experimental use to reduce experimental errors. According to our previous study [17], cellular models of the three types of cells were constructed. The models were grouped into the control (cellular model), M (cellular model + 20 *μ*g/ml freeze-dried powder), and M + D (cellular model + 20 *μ*g/ml freeze-dried powder + DAPT at 10 *μ*M) groups and were each cultured for 48 h.

#### 2.2.3. Observational Indices

1. Western blot analysis of the levels of Notch1, Notch2, *α*-SMA, DLL1, DLL4, Jag1, and Jag2. The collected cells were lysed with 200 *μ*l of radioimmunoprecipitation assay (RIPA) solution for 30 min. After transferal into a prechilled 1.5 ml centrifuge tube (), the lysis solution was centrifuged at 12,000 rpm at 4°C for 10 min. Then, the supernatant was stored at −20°C. The protein concentrations were determined with a BCA kit (Beyotime, China). Gel electrophoresis (sodium dodecyl sulfate (SDS)-polyacrylamide) was conducted, and the Mini-PROTEAN 3 electrophoresis tank (Bio-Rad Laboratories, USA) was used, which was followed by wet transfer with the Trans-Blot SD tank (Bio-Rad Laboratories). After transfer, the membrane was blocked in 5% skimmed milk in TBST buffer. The samples were incubated with primary antibodies and anti-GAPDH (200306-7E4; Zen-bio) at 4°C overnight. Horseradish peroxidase (HRP)-labeled anti-rabbit antibodies (secondary antibodies; A0208; Beyotime) were added for a 1 h incubation at room temperature. Signals were detected with SuperSignal West Pico Chemiluminescent Substrate. The relative levels of the protein bands were quantitated with Bio-Rad ChemiDoc XRS software. The blot images were examined by Image Quant TL software (v2003; Amersham Biosciences, Freiburg, Germany).
2. Biochemical analysis of the levels of the products of oxidative stress. ROS, SOD, GSH, and MDA levels were examined biochemically with an oxidative stress kit (S0033; Beyotime), SOD kit (S0101; Beyotime), GSH-PX kit (A005; Nanjing Jiancheng), and MDA kit (A003-1; Nanjing Jiancheng), respectively. The calculation was performed rigorously following the instructions in the kit.

### 2.3. Statistical Analysis

All the data were analyzed with the SPSS 21.0 software, with the measurement data presented as *x* ± *s*. If the data were normally distributed, ANOVA was performed to compare among the groups, which was followed by the least significant difference (LSD) correction. Otherwise, nonparametric analysis was carried out. A difference of *P* < 0.05 was considered to be significant. Plots were drawn with GraphPad Prism v8.

## 3. Results

### 3.1. General Condition

The rats in the control group were healthy, and they showed normal appetite. In contrast, the activities of rats in the remaining groups gradually reduced. Relative to the control group, both the model and intervention groups showed signs of mental fatigue, coughing, decreased appetite, no weight gain, and delayed responses to external stimuli. The symptoms in the M + D group were mild.

### 3.2. Pulmonary Pathological Changes

**Figure 1:**
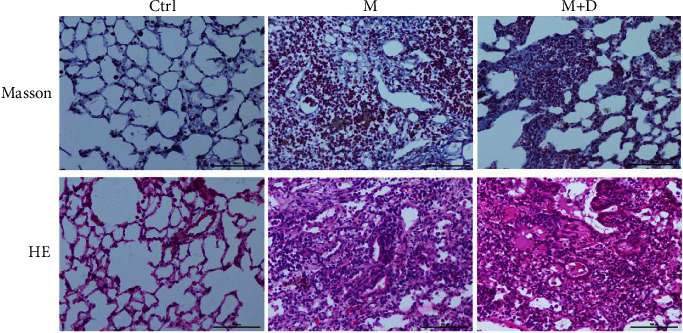
Pathological observation of the rat pulmonary tissues after HE and Masson staining (×200).

According to the outcomes of our prior experiment, the animals were sacrificed at 20 w, and HE and Masson staining were performed (Figure 1). HE staining showed that the control group exhibited a clear alveolar structure with a thin alveolar wall. The samples had no thickened or congested alveolar septa, inflammatory cell infiltration, or bleeding. The M group exhibited collapsed and destroyed alveolar structure and abundant deposited collagen, with collagen and fibrin occupying the alveolar septa and alveoli. The M + D group exhibited a large number of inflammatory cells with granulomatous changes. Masson staining showed that the control group did not have deposited collagen in lung cells. In the M group, plenty of collagen fibers that were stained blue were observed in the alveoli, and there was collagen fiber proliferation in the great vessel wall. Collagen fibers surrounded inflammatory cells with noticeable interstitial fibrosis, with some alveolar septa thickened. Part of alveolar structure disappeared with fibroblasts proliferating around the glands. Fibrous tissue extended in patches. The M + D group exhibited a small amount of deposited collagen.

### 3.3. *α*-SMA Level

**Figure 2:**
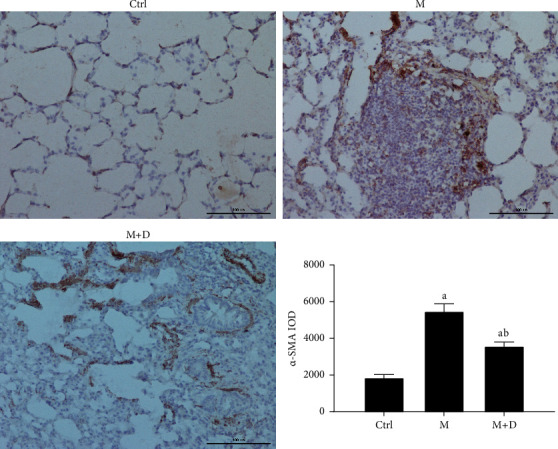
Immunohistochemical analysis of the protein levels of *α*-SMA in different groups (×200). *Notes*. a and b mean *P* < 0.05 vs. the control group and the M group, respectively.

The protein levels of *α*-SMA in the groups were observed using immunohistochemistry (Figure 2). Protein-positive signals were indicated by brownish-yellow granules, and they were primarily located in the pulmonary interstitium. Stronger positive reaction signals indicate a more severe inflammation in the area. Relative to the control group, the remaining groups both showed stronger positive signals (*P* < 0.05), but the number of positive granules in the intervention group (M + D) was noticeably less than the M group. Relative to the control group, the M and M + D groups both had higher *α*-SMA levels (*P* < 0.05), but the levels in the latter group were significantly lower than the former group (*P* < 0.05).

### 3.4. Key Notch Proteins

**Figure 3:**
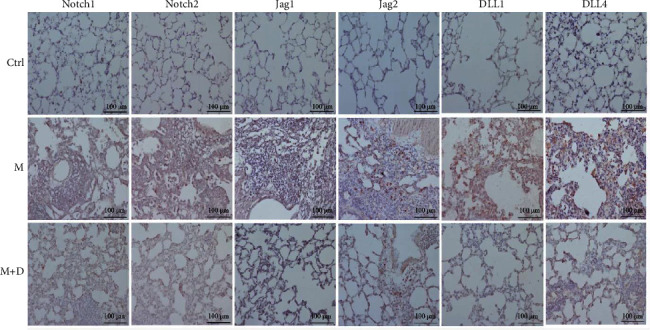
Observation of the key Notch proteins in the pulmonary tissue of the different groups of rats using immunohistochemistry (×200).

**Figure 4:**
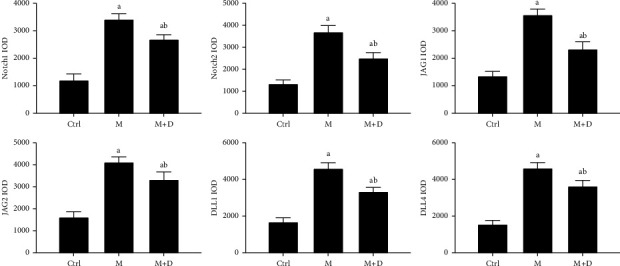
Comparison of the levels of the key proteins of the Notch pathway, i.e., Notch1, Notch2, JAG1, JAG2, DLL1, and DLL4, in the pulmonary tissue of different groups of rats. *Notes*. IOD, integrated optical density; a and b mean *P* < 0.05 vs. the control group and the M group, respectively.

The key proteins of the Notch pathway in rat lungs were analyzed using immunohistochemistry. In Figure 3, the key proteins of the Notch signaling pathway are indicated in the *x* axis, i.e., Notch1, Notch2, Jag1, Jag2, DLL1, and DLL4. In the figure, positive signals were indicated by brownish-yellow granules, which mostly concentrated in the pulmonary interstitium, and stronger positive signals indicated more severe inflammation in the area. Positive signals for the key proteins were consistent across the groups. Relative to the control group, the M group showed markedly stronger signals. Compared with the M group, however, the M + D group had noticeably reduced signals. Consistently, although the M and M + D groups showed significantly higher levels of key proteins than the control group, the M + D group had significantly lower levels of these proteins than the M group (*P* < 0.05; Figure 4).

### 3.5. Oxidative Stress in Pulmonary Fibrosis

**Figure 5:**
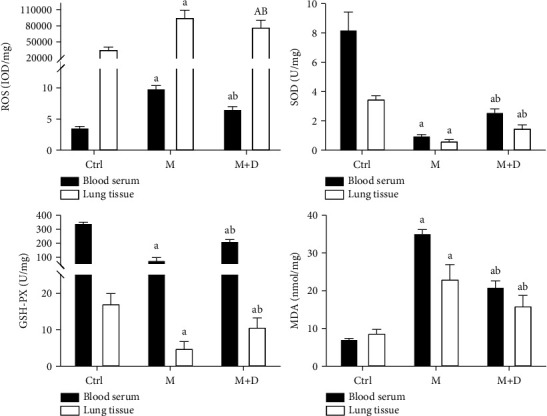
Comparison of the levels of serum and pulmonary oxidative stress products, i.e., SOD, GSH-PX, MDA, and ROS, in different groups. *Notes*. a and b mean *P* < 0.05 vs. the control group and the M group, respectively.

Serum and pulmonary SOD, GSH-PX, MDA, and ROS levels were detected (Figure 5). Relative to the control group, both the M and M + D groups had a significantly higher ROS level (*P* < 0.05), and the M and M + D groups did not show a significant difference (*P* < 0.05). In terms of SOD and GSH-PX, despite significantly lower levels in the M and M + D groups relative to the control, the M + D group had higher levels than the M group (*P* < 0.05). The M and M + D groups also had higher MDA levels than the control (*P* < 0.05), and similarly, the M + D group exhibited a reduced level compared with the M group (*P* < 0.05).

### 3.6. Outcomes of the Cytological Validation Experiments

#### 3.6.1. Notch Signaling Pathway in AECIs during Pulmonary Fibrosis

**Figure 6:**
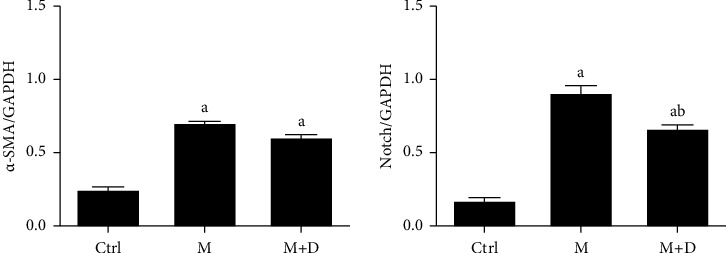
Comparison of the Notch and *α*-SMA levels (relative to GAPDH) in type I alveolar epithelial cells of different groups. *Notes*. a and b mean *P* < 0.05 vs. the control group and the M group, respectively.

The Notch pathway and *α*-SMA levels in AECIs were determined (Figure 6). The M and M + D groups exhibited significantly increased *α*-SMA compared with the control group (*P* < 0.05), but there was no significant difference between the M and M + D groups (*P* > 0.05). Compared with the control group, the M group and the M + D group both showed increased Notch levels (*P* < 0.05), and significantly decreased Notch levels were exhibited in the M + D group relative to the model group (*P* < 0.05).

#### 3.6.2. Notch Signaling Pathway in Type II Alveolar Epithelial Cells

**Figure 7:**
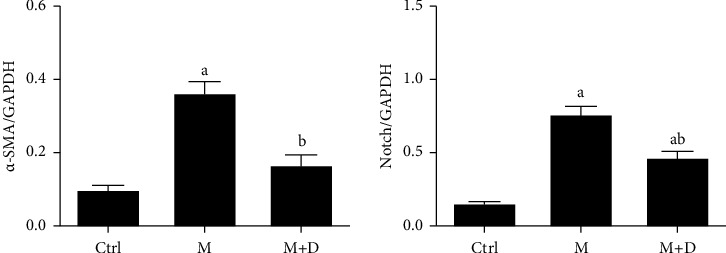
Comparison of the Notch and *α*-SMA levels (relative to GAPDH) in type II alveolar epithelial cells of different groups. *Notes*. a and b mean *P* < 0.05 vs. the control group and the M group, respectively.

Results of the Notch and *α*-SMA levels in AECIIs are shown in Figure 7. Relative to the control group, the M group showed significantly increased *α*-SMA (*P* < 0.05), while the M + D group showed decreased *α*-SMA as compared to the M group (*P* < 0.05). Relative to the control group, both the M and M + D groups showed increased Notch levels (*P* < 0.05), and significantly decreased Notch levels were exhibited in the M + D group relative to the M group (*P* < 0.05).

#### 3.6.3. Notch Levels in Fibroblasts

**Figure 8:**
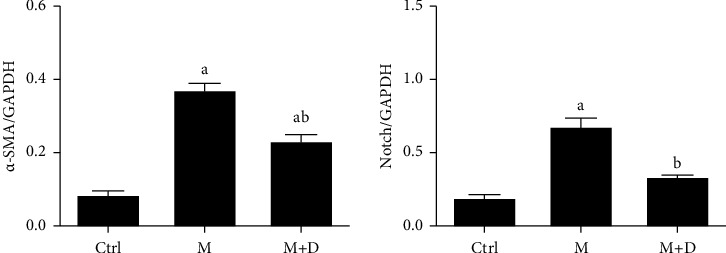
Comparison of the Notch and *α*-SMA levels (relative to GAPDH) in fibroblasts of different groups. *Notes*. a and b mean *P* < 0.05 vs. the control group and the M group, respectively.

The levels of the Notch pathway and *α*-SMA in fibroblasts were detected (Figure 8). Relative to the control, the M group showed significantly increased *α*-SMA levels (*P* < 0.05), and the M + D group showed decreased *α*-SMA levels relative to the M group (*P* < 0.05). In comparison with the control, both the M and M + D groups showed significantly increased Notch levels (*P* < 0.05), and significantly decrease was observed in the M + D group compared with the model group (*P* < 0.05).

#### 3.6.4. Oxidative Stress Regulation by the Notch Pathway in AECIs

**Figure 9:**
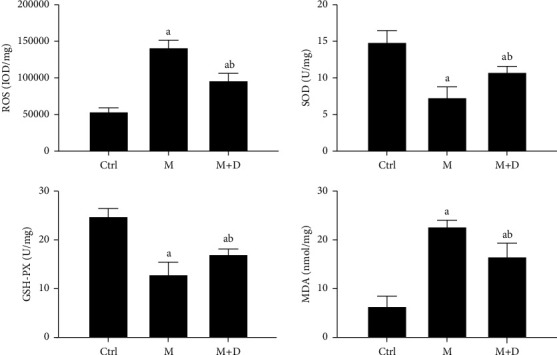
Comparison of the levels of oxidative stress products, i.e., ROS, SOD, GSH-PX and MDA, in type I alveolar epithelial cells of different groups. *Notes*. a and b mean *P* < 0.05 vs. the control group and the M group, respectively.

The ROS, SOD, GSH-PX, and MDA levels in AECIs were determined (Figure 9). Both the M group and the M + D group showed increased ROS levels relative to the control group (*P* < 0.05), but the M + D group had decreased ROS compared with the model group (*P* < 0.05). Although the M and M + D groups showed lower levels of SOD and GSH-PX than the control, the M + D group showed higher levels than the model group (*P* < 0.05). In terms of MDA, both the M group and the M + D group had a significantly higher level relative to the control (*P* < 0.05) and the M and M + D groups significantly differed (*P* < 0.05).

#### 3.6.5. Oxidative Stress Regulation by the Notch Pathway in Type II Alveolar Epithelial Cells

**Figure 10:**
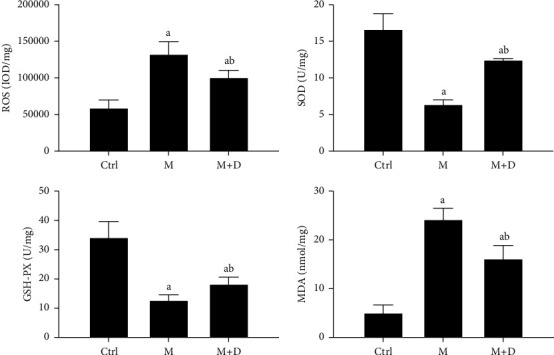
Comparison of the levels of oxidative stress products, i.e., ROS, SOD, GSH-PX and MDA, in type II alveolar epithelial cells of different groups. *Notes*. a and b mean *P* < 0.05 vs. the control group and the M group, respectively.

The ROS, GSH-PX, SOD, and MDA in AECIIs were detected, and the results are shown in Figure 10. The M and M + D groups exhibited a higher ROS level relative to the control (*P* < 0.05), and the M + D group had significantly decreased ROS levels relative to the model group (*P* < 0.05). The M and M + D groups had significantly lower levels of GSH-PX and SOD than the control, and the M + D group showed higher levels than the M group (*P* < 0.05). In terms of MDA, the M and M + D groups had a higher level as compared to the control (*P* < 0.05), and a decrease was found in the M + D group relative to the model group (*P* < 0.05).

#### 3.6.6. Oxidative Stress Regulation by the Notch Pathway in Fibroblasts

**Figure 11:**
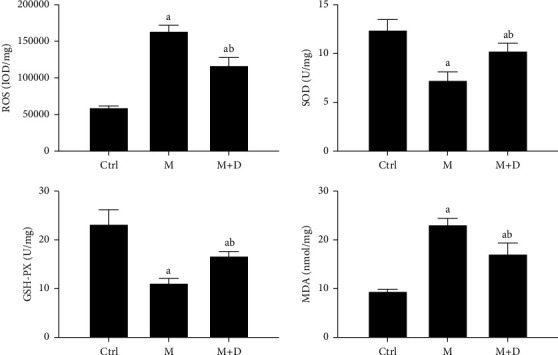
Comparison of the levels of oxidative stress products, i.e., ROS, SOD, GSH-PX and MDA, in fibroblasts of different groups. *Notes*. a and b mean *P* < 0.05 vs. the control group and the M group, respectively.

The ROS, GSH-PX, SOD, and MDA levels in fibroblasts are shown in Figure 11. Although the M and M + D groups had a higher ROS level relative to the control group (*P* < 0.05), the M + D group had a significantly decreased ROS level as compared to the M group (*P* < 0.05). The M and M + D groups also had significantly lower GSH-PX and SOD levels relative to the control group, the levels in the M + D group were significantly higher than those in the M group (*P* < 0.05). Both the M and M + D groups had a higher level of MDA than the control (*P* < 0.05). A decrease was found in the M + D group relative to the model group (*P* < 0.05).

## 4. Discussion

HP is a highly symptomatic and immune-mediated allergic disease [18]. A variety of factors can lead to this condition, and the pathogenic factors that have been discovered to date include but are not limited to fungi, aerosols, microorganisms in dust, bacteria, and reactive chemicals. Among the various types of HP, PBL/bird fanciers' lung (BFL) is the most common and is caused by environmental factors; it is an allergic disease of the lung in susceptible populations that is induced by long-term inhalation of aerosols formed by bird droppings [19]. Pigeons are a kind of bird, and the composition of respiratory disease in Xinjiang exhibits distinctive regional characteristics. The incidence rate of PBL is high as a joint consequence of the vast territory of Xinjiang, a cold and dry climate, frequent sandstorms, and pigeon breeding as the main economic pillar in some regions [20].

In the Notch signaling pathway, Notch receptors (Delta-like (DLL)) and their ligands (Jagged (Jag)) jointly activate the pathway and promote cellular differentiation and proliferation [9, 21]. When DLL or Jag proteins bind, the internal part of the Notch receptor is cleaved and released into the cytoplasm, which is further cleaved into an active form, thereby activating the Notch signaling pathway [22]. After the Notch signaling pathway is activated, the transcription factor CSL (CBF-1, Suppressor of hairless, Lag collectively) can bind to the active Notch subunit and induce the transcription of downstream genes of the Notch pathway to participate in processes, such as fibroblast proliferation, extracellular matrix synthesis, and epithelial-mesenchymal transition [23, 24]. In this study, we used pigeon feathers and other droppings to prepare freeze-dried protein powder as an allergen, with which we constructed a rat model of fibrotic PBL using the airway instillation method. According to our previous experiment [17], Masson staining was performed on the lung tissue in each group of rats at 20 w. The results showed no significant change in the control group, while the remaining groups exhibited fibrotic manifestations, such as alveolar structure destruction and collagen fiber deposition. However, the manifestations in the M + D group were less severe than those in the M group. Additionally, the protein level of the pulmonary fibrosis marker *α*-SMA in the M + D group was lower than that in the M group. Inhibiting Notch signaling activation can slow the process of pulmonary fibrosis and alleviate the impact of fibrosis on pulmonary function [25]. Our results were consistent with those reported in the literature.

The key Notch proteins and *α*-SMA were examined in this study. After the Notch pathway was inhibited, *α*-SMA levels were not significantly changed in AECIs but were significantly reduced in AECIIs and fibroblasts relative to the model group. Furthermore, the levels of the Notch signaling receptors and ligand proteins in the M group significantly increased when compared with the control and M + D groups. These findings suggested that Notch signaling-associated proteins were highly expressed in fibrotic rats, and activation of the Notch channel is associated with the occurrence of pulmonary fibrosis, which is consistent with the findings reported by another rat study conducted by Guseh et al. [26]. In pulmonary fibrosis tissues, the activity of the Notch pathway is significantly enhanced, and the expression of the Notch signaling pathway regulator Hes1 is increased, which can cause a linkage reaction between fibroblasts and myofibroblasts; this reaction promotes the transformation of myofibroblasts, thereby promoting pulmonary fibrosis [27]. In this study, however, *α*-SMA did not change significantly in type I alveolar epithelial cells after the Notch pathway was inhibited, which may be related to the fact that type I alveolar epithelial cells are mainly responsible for gas exchange and do not transform into cells that are able to produce extracellular matrix. Therefore, inhibiting the Notch signaling pathway may not have a significant impact on the expression of *α*-SMA in type I alveolar epithelial cells [28].

Oxidative stress refers to a pathological imbalance in oxidation-reduction reactions that is caused by excessive oxides generated by metabolism (the generated oxides exceed the clearance capacity). Oxidative stress is the cumulative effect of highly active oxidative molecules, and cell damage is a common outcome of oxidative stress. After oxidative stress in lung tissue is abnormally activated, pulmonary fibrosis can occur. Compared to other organs, lung tissue is more likely to be exposed to higher concentrations of oxygen and is therefore more susceptible to oxidative stress damage [29]. ROS are a normal product of the oxidation-reduction reaction in vivo and can be generated by the normal metabolism of phagocytes, epithelial cells, and neutrophils or induced by external factors, such as smoking, radiation, and dust [30]. ROS can trigger lipid peroxidation, leading to DNA-strand breakage, and indiscriminately oxidize all molecules in biofilms and tissue, thereby leading to damage. When excessive ROS production exceeds the scavenging capacity of the antioxidant system, antioxidant levels decrease, or both conditions coexist and oxidative/antioxidant imbalance can occur, which leads to oxidative stress [31]. ROS can also lead to apoptosis in AECs through the Notch and other pathways, participating in the occurrence of pulmonary fibrosis [32–34]. MDA is the final product of lipid peroxidation; changes in its expression can indirectly reflect the degree of peroxidation caused by reactive oxygen species [35]. Under normal conditions, ROS, MDA, and other oxidative stress products can be quickly cleared by SOD, GSH-PX, autophagy, and other antioxidant defense systems. These factors do not necessarily pose a threat to the body under physiological conditions [36].

However, when the overproduction of ROS and MDA exceed the clearance capacity of the body or when SOD and GSH-PX are reduced and autophagy is weakened, oxidative/antioxidant imbalance can occur, which leads to oxidative stress damage [37] and pulmonary fibrosis. In this study, we detected the oxidative stress products ROS, MDA, GSH-PX, and SOD. Relative to the model group, both the control group and the M + D group exhibited decreased levels of oxidative stress products and increased levels of oxidoreductase. This finding was consistent with that reported by Khan et al. [38]. The underlying mechanism is presumably as follows: Abnormally activated Notch signaling regulates the expression of NADPH oxidase 4 (Nox4), which further affects ROS production; the overproduction of ROS leads to cell death, thus affecting the occurrence of pulmonary fibrosis [39]. Regulating the Notch channel can affect the activity of GSH-PX and SOD, which are important antioxidant enzymes that can scavenge free radicals in the body [40].

In vitro cultured AECs can undergo epithelial-mesenchymal transition (EMT) through the activation of Notch and other signaling pathways and become a partial source of myofibroblasts (MFBs); MFBs can express the specific biomarker *α*-SMA; this protein is involved in contraction and migration and the excessively secreted extracellular matrix (ECM), which causes the deposition and accumulation of ECM and ultimately leads to the progression of pulmonary fibrosis [41]. In addition, alveolar epithelial cells and bronchial epithelial cells can be transformed into MFBs through the EMT pathway; in lung biopsy tissue samples of IPF patients, EMT has been shown to be associated with the occurrence of pulmonary fibrosis [42]. In this study, we cultured AECIs, AECIIs, and fibroblasts in vitro and determined the protein levels of the Notch channel and *α*-SMA. After the cells were stimulated with allergens, the key Notch protein and *α*-SMA levels were significantly increased. This change may be associated with specific *α*-SMA expression in some alveolar epithelial cells during their transformation into myofibroblasts. In addition, Notch signaling is associated with other cellular functions during pulmonary fibrosis, such as the destruction of alveolar epithelial cells, ECM deposition, and fibroblast formation, which are all important characteristics in pulmonary fibrosis development. EMT is a crucial link in the occurrence of pulmonary fibrosis; various cytokines and signaling pathways interact to form a network, and these interactions affect the occurrence of pulmonary fibrosis [43]. Activation of the Notch signaling pathway can enhance the cellular apoptosis cascade, which can directly or indirectly cooperate with other signaling pathways to induce EMT [44], thereby affecting the development of pulmonary fibrosis.

To further explore the molecular mechanism behind the participation of the Notch signaling channel in pulmonary fibrosis in PBL by regulating oxidative stress, we further determined the SOD, GSH-PX, MDA, and ROS protein levels in cultured cells, and the relationships among the Notch pathway, oxidative stress, and pulmonary fibrosis were examined. After the Notch pathway was inhibited by DAPT, the levels of ROS and MDA decreased, while GSH-PX and SOD levels increased relative to those in the model group. According to our findings, the expression of ROS was consistent with that of MDA and was associated with negative regulation; that is, the increased levels suggested more severe pulmonary fibrosis. The expression of GSH-PX was consistent with that of SOD and was associated with positive regulation, suggesting that the increased levels indicated less severe pulmonary fibrosis and corresponding changes in Notch.

The detection of Notch-related proteins and oxidative stress levels in the three types of cells further demonstrated that Notch and oxidative stress were involved in the development of pulmonary fibrosis in PBL. When excessive oxidative stress products exist, related pathways are activated, which affect cell orientation and further promote the release of various inflammatory mediators and cytokines. These mediators and cytokines include TGF-*β* production. An increase in TGF-*β* can further promote the release of mitochondrial ROS in cells and further promote EMT, fibroblast differentiation, and apoptosis, thereby promoting the occurrence and development of pulmonary fibrosis. Matsuzawa et al. [45] showed that oxidative stress levels were elevated in patients with IPF by measuring the levels of ROS and MDA; among IPF patients whose condition had not yet been controlled, the level of oxidative stress was significantly increased compared to those whose condition had been controlled, indicating an imbalance between oxidation and antioxidants during the progression of IPF. Our results are consistent with theirs.

There are some limitations in this study. First, there are certain differences in the pathogenesis of animals and humans, and therefore, the findings obtained on animals may not be applicable to humans. The findings of this study remain to be validated by clinical trials in the future. Second, the sample size of this study was small, and therefore, studies with larger sample sizes remain to be conducted to further validate the findings of this study. Third, the Notch signaling pathway was inhibited by DAPT in this study; that is, the Notch pathway was blocked by selectively inhibiting *γ*-secretase. However, the specific roles of key downstream proteins have not yet been effectively determined, which might cause bias to the results of this study to some extent. Thus, further research on the key genes related to the receptors and ligands is required. Fourth, in the cytological experiment, we were unable to simulate the normal signaling pathway in the body, and only intervention and observational research was carried out, for which the experimental results might be biased. Fifth, during type II alveolar epithelial cell subculture, the cells are prone to undergo differentiation, rapid denaturation, and rapid phenotype loss, which make cell subculture almost impossible. Therefore, we purified the cells after isolation, which resulted in differences from normally subcultured cells. Also, we performed separate intervention studies for each cell model and did not conduct research on the mechanisms of Notch signaling and oxidative stress based on the interactions among AECIs, AECIIs, and fibroblasts, which would constitute an important research direction in the future. Last, three types of cell models were involved in this study for results verification, which was performed after 4 h culture. Throughout this study, we did not investigate the long-term effect of the Notch pathway inhibitor, which constituted another limitation of this study, as well as an important direction for future research.

## 5. Conclusion

In conclusion, Notch signals and oxidative stress play essential roles in pulmonary fibrosis in PBL, and interactions among Notch, oxidative stress, and pulmonary fibrosis in target cells exist. Furthermore, this study preliminarily revealed the effect of intervening in the Notch signaling channel on oxidative stress and pulmonary fibrosis. Based on the findings of this study, future research can be performed on how the Notch signaling pathway affects the molecular network within cells, what the interactions between Notch pathway and other signaling pathways (e.g., TGF-*β* and Wnt) are like, as well as how these interactions jointly promote the development of pulmonary fibrosis. The findings of this study may provide theoretical basis as well as a molecular target for the clinical treatment of pulmonary fibrosis in PBL.

## Data Availability

Data analyzed in this study are available from the corresponding author CW on reasonable request.
